# Voluntary wheel running in dystrophin-deficient (mdx) mice: Relationships between exercise parameters and exacerbation of the dystrophic phenotype

**DOI:** 10.1371/currents.RRN1295

**Published:** 2012-03-19

**Authors:** Gayle M Smythe, Jason D White

**Affiliations:** ^*^School of Community Health and Centre for Inland Health, Charles Sturt University and ^†^Murdoch Childrens Research Institute and School of Veterinary Science, University of Melbourne

## Abstract

Voluntary wheel running can potentially be used to exacerbate the disease phenotype in dystrophin-deficient mdx mice. While it has been established that voluntary wheel running is highly variable between individuals, the key parameters of wheel running that impact the most on muscle pathology have not been examined in detail. We conducted a 2-week test of voluntary wheel running by mdx mice and the impact of wheel running on disease pathology. There was significant individual variation in the average daily distance (ranging from 0.003 ± 0.005 km to 4.48 ± 0.96 km), culminating in a wide range (0.040 km to 67.24 km) of total cumulative distances run by individuals. There was also variation in the number and length of run/rest cycles per night, and the average running rate. Correlation analyses demonstrated that in the quadriceps muscle, a low number of high distance run/rest cycles was the most consistent indicator for increased tissue damage. The amount of rest time between running bouts was a key factor associated with gastrocnemius damage. These data emphasize the need for detailed analysis of individual running performance, consideration of the length of wheel exposure time, and the selection of appropriate muscle groups for analysis, when applying the use of voluntary wheel running to disease exacerbation and/or pre-clinical testing of the efficacy of therapeutic agents in the mdx mouse.

## Introduction

Duchenne muscular dystrophy (DMD) is a prevalent neuromuscular disease resulting from a mutation affecting the dystrophin gene, and resulting in widespread deficiency of the muscle membrane protein, dystrophin [1] [2]. This causes a loss of membrane integrity, and muscle susceptibility to damage and degeneration. The mdx mouse is a widely used animal model for understanding and testing of potential therapeutic agents in DMD [3] [4]. However, the applicability of this rodent model to the human disease situation is considered limited because the extent of muscle pathology is milder, and the pattern by which it occurs and progresses differs, to that in humans with DMD [3]. It has been suggested that the mdx dystrophic phenotype can be exacerbated to more closely resemble human DMD pathology by exposing animals to an exercise regime. Exercise regimes have now been utilized to exacerbate the dystrophic phenotype and allow investigation of key characteristics and mechanisms of muscle weakness and degeneration [5], and to increase the rigor of pre-clinical testing of therapeutic agents [6] [7] [8] [9] [10].   

Exercise-induced exacerbation has been trialed in a number of forms, including the use of treadmills, swimming, and voluntary wheel running [4]. Of these, voluntary wheel running is the most convenient, cost- and time-effective, and the least invasive. However, there is controversy as to the impact of voluntary wheel running regimes on muscle structure and function in the mdx mouse. Some studies have reported that voluntary wheel running has beneficial effects on mdx muscle [11] [12] [13] [14] [15], while others have demonstrated detrimental effects [16]. For the most part, these studies have relied on the use of either total cumulative or daily distances run on average for all mdx mice as an indicator of overall performance. In addition, there is clearly variation in study design, with some utilizing only a very short, acute bout of exercise [16], while others allow longer-term access to running wheels [12] [13] [14] [15]. This may be a problematic approach based on recent studies that clearly show a high level of variation between individual animals in their willingness and performance on voluntary wheel running over a 48 hour period [9] [17]. To determine if such variability continues over a longer period, we analyzed individual mdx mouse performance on voluntary wheels over a two-week period. Furthermore, we hypothesized that there is complexity in the parametric determinants of muscle histopathology in mdx mice undertaking voluntary wheel running. To test this hypothesis, we undertook a detailed correlation analysis to identify key indicators of running activity for exercise-induced exacerbation of the dystrophic phenotype. 

## Materials and Methods

### Animals

Twenty-one adult male mdx mice, aged 10-12 weeks, were obtained from the Animal Resources Centre (Perth, WA). Mice were housed in standard cages with food and water freely available, in a clean, well-ventilated room with an ambient temperature set at 23°C. Room lighting was set on a 12-hour day/night cycle. Of the 21 mice, 10 constituted the non-exercised (sedentary) controls, while 11 were given free 24-hour per day access to an exercise wheel. Prior to the commencement of the running trial, the mice in the exercise group were placed in a cage containing an exercise wheel for 7 days. During this time mice became familiar with the wheel, and a calibration of the wheel system carried out to determine the most appropriate time periods for logging wheel rotation data. The calibration involved collecting wheel rotation data while filming mouse behavior. Parallel analysis of rotation counts with behaviour indicated that logging wheel rotation data every 30 seconds, and including logged data for 2 or more subsequent 30 second periods, was the most appropriate method of reducing false data being included as a result of wheel play (where mice stood outside the wheel and moved it with their forelimbs). The filming of mouse behavior also demonstrated that the wheels did not register further rotations after mice either stopped wheel play or exited the wheel after a run bout. The 11 mice in the exercise group were then placed in separate cages, for collection of individual running data. All cages were in both visual and hearing distance from one another, spaced approximately 10 centimetres apart. All animal procedures were carried out in accordance with the guidelines of the National Health and Medical Research Council of Australia, and with the approval of the institutional ethics committee (University of Melbourne).   

### Exercise wheel design

Standard mouse exercise wheels were attached directly to the side wall of the cages; each wheel had a magnet directly attached. Embedded in PVC at the wheel-cage connection point were 4 micro Read switches spaced at 90^o^ to each other, allowing for detection and recording of every quarter turn of the wheel. As the magnet rotated past each switch the magnetic field closed the switch, thus closing the circuit. Data were collected and stored on PICAXE20 microprocessors. The microprocessors were programmed to only record a quarter of a rotation after a second switch was recorded as closed; this reduced the impact of wheel play. Data from the microprocessors were downloaded at a time interval which is set by the software. For the purposes of this study, data were downloaded every 30 seconds.  

### Tissue sampling and staining

On the day of sampling, mice were euthanized by brief halothane inhalation, followed by cervical dislocation. Quadriceps femoris, gastrocnemius (medial heads) and tibialis anterior muscles were immediately dissected out, bi-sected transversely through the muscle belly, embedded in Tragacanth gum (Sigma, Australia) on cork blocks with the cut surfaces at the block face, and snap-frozen in liquid nitrogen-cooled isopentane. Sections (8 µm) were cut on a standard cryostat, mounted on glass microscope slides, and stained with haematoxylin and eosin according to standard methodology. Glass coverslips were mounted with DePex mounting medium (ProSciTech, Australia), and sections viewed with an Olympus CX41 light microscope. Digital images were obtained with a Luminera Infinity digital camera (HD Scientific, Australia). Full cross-section images through the muscle were obtained by acquiring multiple images at low power (2.5x objective lens) and aligning them manually using Powerpoint software (Microsoft).   

### Wheel running data collection

Wheel running data were directly logged to a spreadsheet (Microsoft Excel) as outlined above. Daily distances run by individual mice were calculated from the trial start time, in 24-hour periods, with the first day of exercise designated as day 1. Detailed analyses of wheel use were limited to data collected during the 12-hour overnight (lights off) period as the bulk of running occurred during the dark hours in accordance with previous studies (Briguet et al. 2004; see also Fig. 1C). Mean daily and cumulative total distances were determined in kilometres by direct conversion of recorded revolution counts. Detailed patterns of wheel use were analyzed for each mouse, for the entire 12-hour overnight period on all nights of the two-week test period. These data provided moment-to-moment changes in wheel use, with running bouts determined as periods in which wheel revolution counts were recorded in two or more subsequent 30-second recording periods. For each running bout, the distance covered and rate of running were calculated, to provide values for mean running bout distance, and mean running rate. Rest times were designated as periods between running bouts where no revolution counts were recorded.   

### Histopathological analyses

Histopathological analyses were conducted in quadriceps and gastrocnemius muscles taken from one limb of all control mice (n=10 for each muscle group). For two of the 11 exercised mice (mouse 1 and mouse 5), muscles from both limbs were available for analysis (n=13). To account for variation resulting from the focal nature by which necrotic damage typically occurs in mdx muscle, the percentage of necrotic tissue was determined in the entire cross-section of each quadriceps muscle. Necrotic tissue was classified as areas in which few or no intact myofibers or newly formed myotubes were present, and there was clear infiltration by inflammatory cells. Low power images were overlaid to produce a single image of a whole cross-section through the muscle (see Fig. 5). The percentage of necrotic tissue was quantified using a geographical information systems program, ARCMap10 (ArcGIS). Using this software, images were opened, and necrotic areas, and the whole cross-sectional area, were traced using the free-hand polygon drawing tool and their areas calculated using arbitrary units. The combined area of all necrotic areas was then calculated as a percentage of the total cross-sectional area.   

The percentage of centrally nucleated myofibers was determined by obtaining 10 random, non-overlapping images from a single tissue section, using a 10x objective lens, and recording the total number of myofibers, and the number of these that were centrally nucleated. For each tissue section, the data from the 10 images were combined to determine the mean percentage of centrally nucleated fibres. The mean number of myofibers counted for each muscle was 173 ± 96, with variability due to random selection of images and the extent of fiber sizes.   

The mean myofiber cross-sectional area (CSA) was derived from Feret’s minimal cross-sectional diameter after using immunohistochemical staining for laminin to define the myofiber perimeter, according to standard methodology [18]. The CSA for all myofibres in the cross-section was determined.   

### Data analysis

Pearson correlation coefficients were calculated using SPSS software, and deemed statistically significant at the 0.05 level. Differences between exercised mice and sedentary controls for histopathological parameters were determined using Student’s t-tests, with differences deemed significant at the 95% confidence level (p<0.05). Differences between individual exercised mice in mean daily running distance and running rates were determined using one-way ANOVA and the least significant difference post-hoc test with SPSS software.   

## Results

To first gain an overview of exercise wheel use, average daily distances were calculated for each mouse. These values varied significantly between individual mice (Fig. 1A). The average daily distance run by mouse 7 (0.003 ± 0.005 km) and 8 (0.55 ± 0.50 km) was significantly less than that for all other mice (Fig. 1A). Mouse 4 also ran a relatively low daily distance (1.83 ± 0.86 km) and this differed significantly to all except mice 1 and 2. Mice 9-11 all ran on average > 4 km per day, while mice 1-3, 5 and 6 all ran intermediate daily distances (ranging from 2.49 ± 1.49 km (mouse 1) to 3.79 ± 1.53 km (mouse 5)) (Fig. 1A). We also compared the cumulative distance run over the whole test period for each mouse. Mouse 10 (67.24 km) and 11 (62.68 km) ran the furthest total distances, while mouse 7 (0.04 km) and 8 (7.65 km) ran the shortest distances (Fig. 1B). A plot of the daily running activity for each mouse throughout the trial clearly demonstrated the extent of variability between individuals, although for all individuals the bulk of voluntary wheel use was during the dark hours (Fig. 1C). Interestingly, the daily distances run by individuals diverged more over time, with the range of individual daily distances varying (difference between highest performance and lowest performance) by 4.26 km on day 1, 4.94km on day 7 and 6.65 km on day 14 (Fig. 1C).  

**Figure:**
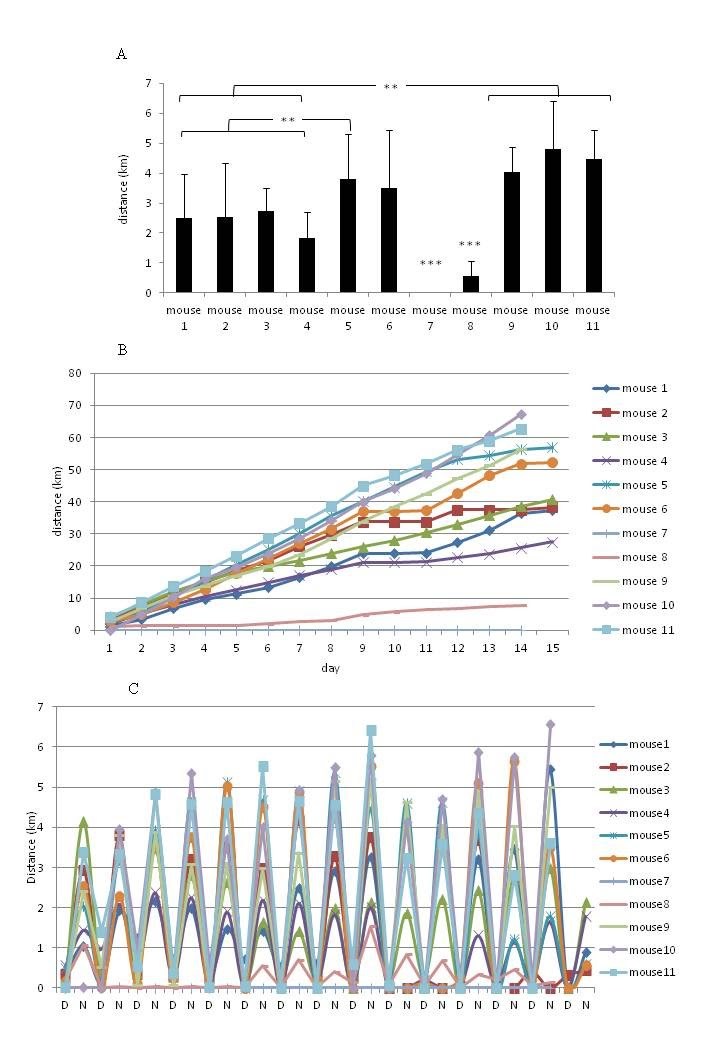
Figure 1. Overview of running data for all mice. (A) Average daily running distance for individual mice. Values shown are mean daily distance (km) ± standard deviation. Statistical comparisons were made between all individuals (Student’s t-test, *p<0.05; **p<0.005; ***p<0.0005). (B) Cumulative daily distances run by individual mice. (C) Plots of individual running patterns according to the day/night light cycles (day (D) = lights on (12 hours); night (N) = lights off (12 hours)). 

To assess changes in running behavior over time, a detailed analysis of wheel running for individual mice was conducted on all nights. Running behavior during the 12 hour dark cycle only was analyzed because our data (see Fig 1C) and those of others [14] [15] [17] [19] clearly demonstrate that mouse voluntary wheel running (irrespective of mouse strain) predominantly occurs at night. There were striking differences between individual mice in performance (Fig. 2). Mice 1-4, 7 and 8 showed a consistent pattern of intermittent running and resting throughout most of the dark hours (Fig. 2A, mouse 3 shown). Mice 5 and 10 similarly ran relatively high overnight distances, but this was achieved by running through most of the night, albeit with more regular non-running (rest) breaks in the last 4-6 hours of the night (Fig. 2B, mouse 10 shown). Mice 6, 9 and 11 also ran intermediate-high overnight distances, although these were variable by up to 3 km between nights; interestingly, these mice largely ran in the first 6 hours of the night, with little or no running activity in the final 6 hours (Fig. 2C, mouse 11 shown). 

**Figure:**
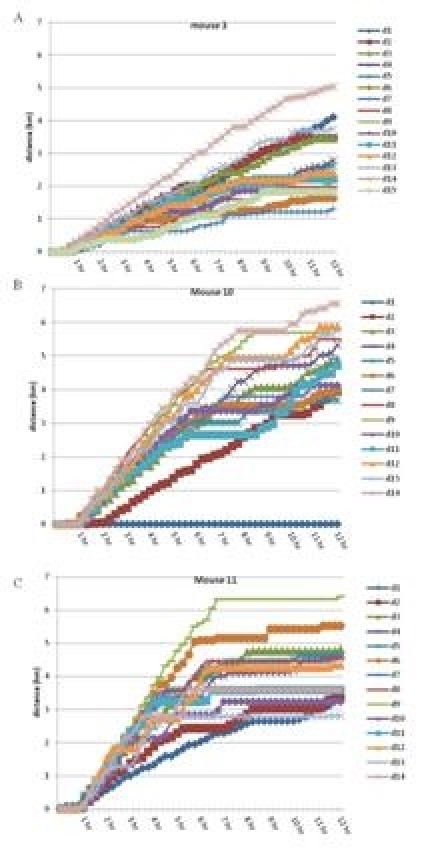
Figure 2: Plots of overnight cumulative distances for representative mice on all days. There was extensive variability in individual performance both between and within each day examined. Some mice, such as mouse 3 (A) ran a broad range of overall distances (1-5km), and ran a consistent pattern of short bursts of running interspersed with non-exercise periods. Others such as mouse 11 (B) showed less variability between days in overall distance, ran greater distances (3-6km), and ran almost exclusively in the first 6 hours of the night. In contrast, mouse 10 (C) also ran very high and less variable distances (4-6.5km), and ran throughout the night.

A detailed analysis of moment-to-moment changes in wheel use was conducted for all mice, on all nights of the study. The average time spent on the running wheel per running bout, which was defined as occurring when wheel revolutions were recorded in two or more subsequent 30-second periods, ranged from 1.04 minutes in mouse 7, to 10.52 minutes for mouse 11 (Fig. 3A). Wheel running bouts were significantly shorter for mice 4, 7 and 8 (p<0.005; Fig. 3A), and significantly longer for mouse 11 compared with all others (p<0.05, Fig. 3A). In contrast, rest times between running bouts did not differ between most individuals, with the exception of mice 7 and 11 who had mean rest times that were significantly higher than for all other mice (p<0.005; Fig. 3B). The average speed (m/min) and the total number of running bouts were also determined for the 2-week running period for all mice (Fig. 3C). In relation to average running rate, mice fell into 5 groups that differed significantly from one another (p<0.05) being, from slowest to fastest, mice 7 and 8, mice 1 and 4, mice 2 and 3, mice 5 and 6, and mice 9-11 (Fig. 3C). The total number of running bouts (also shown in Fig. 3C) combined with the average running speed gives an indication of running efficiency. Mouse 8 ran a very high number of bouts but at a very slow average rate, while at the other extreme, mice 6 and 9-11 ran relatively few bouts at a very fast average rate. 

**Figure:**
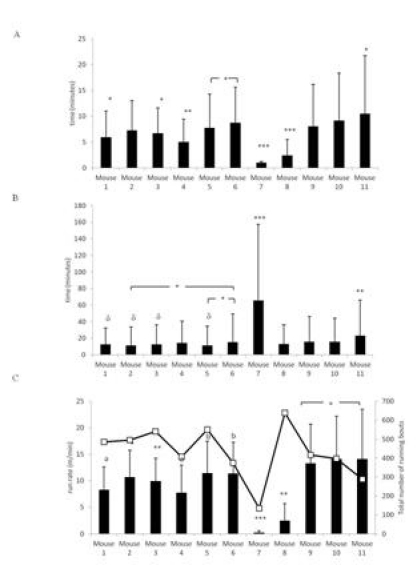
Figure 3: Analysis of (A) running bout time, (B) rest (non-exercise) time, and (C) running rate and number of running bouts. All values shown are mean ± standard deviation, in minutes for A and B. In C, the bars shown are mean run rate (left vertical axis) ± standard deviation, in metres/minute (m/min), while the line represents the total cumulative number of running bouts in the entire trial (right vertical axis). (A) Mice 1, 3, 4, 7 and 8 had a significantly shorter mean running time compared with all other mice, while mouse 11 had a significantly longer mean running time (*p<0.05, **p<0.005, ***p<0.0005). (B) Mice 7 and 11 had mean rest times that differed significantly to all other mice (**p<0.005, ***p<0.0005), while mice 1, 2, 3 and 5 had a mean rest time that was significantly lower than mice 9 and 10 (*p<0.05). (C) The mean run rate for mice 2, 3, 7 and 8 differed significantly to all other individuals (*p<0.05, **p<0.005, ***p<0.0005), and that for mice 9-11 was significantly faster than all other mice (*p<0.05).  The mean run rate for mice 1 and 4 differed to all except each other (ap<0.05), and for mice 5 and 6 also differed to all except each other (bp<0.05). 

To determine which muscle/s are most affected by exercise, we examined the histopathology of the quadriceps and gastrocnemius from all mice, using three parameters; the percentage of tissue necrosis, percentage of centrally nucleated myofibers, and the myofiber cross-sectional area (CSA). The tibialis anterior muscle was also examined, but as there was little evidence of individual variation or an impact of exercise on muscle architecture (data not shown), this muscle group was excluded from further study. There was a significant increase in tissue necrosis in the quadriceps muscles from exercised mice (Fig. 4A). While there was no significant change in the percentage of necrosis in the gastrocnemius muscles from exercised versus sedentary mice, the level of individual variability was striking, particularly in the exercised group (Fig. 4A). In both muscle groups, the percentage of centrally nucleated myofibers was very high (approximately 80%) prior to exercise, and this increased slightly with exercise in the quadriceps but not the gastrocnemius muscles (Fig. 4B). Due to the cyclic damage/repair process that occurs in dystrophic muscle, myofiber CSA was highly variable in quadriceps and gastrocnemius muscles in both sedentary and exercised mdx mice, and testing of mean values demonstrated no significant differences between muscles or treatment groups (data not shown). A histogram of myofiber CSA demonstrates that the gastrocnemius muscles from both sedentary and exercised mice had a higher percentage of very small (<100µm^2^) and very large (>4000µm^2^) myofibers compared with the quadriceps muscles (Fig. 4C). In the quadriceps muscle, wheel running caused a slight shift towards larger myofiber CSA as indicated by a decrease in the percentage of small (<500 µm^2^) and a corresponding increase in myofibers in the 1000-3000 µm^2^ size range (Fig. 4C). A similar pattern was observed for the gastrocnemius muscle, although in this case the shift was towards even larger fibers (2000-4000 µm^2^). 

**Figure:**
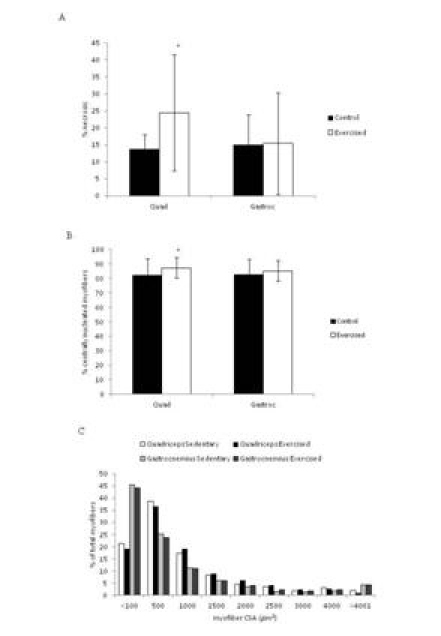
Figure 4: Mean data for quadriceps and gastrocnemius muscles from sedentary control mdx mice versus exercised mdx mice, for (A) the percentage of muscle necrosis, and (B) the percentage of centrally nucleated myofibers. Values shown are mean ± standard deviation. Student’s t-tests were used to determine differences between the control and exercised groups (*p<0.05, **p<0.005), and between the two muscle groups (**p<0.005). (C) Histogram showing the percentage distribution of myofiber cross-sectional area for the quadriceps and gastrocnemius muscles from sedentary and exercised mdx mice. 

Further examination of muscle histopathology showed that, while quadriceps muscles from exercised mice typically had a higher percentage of necrotic tissue compared with the sedentary control mice, variability occurred among exercised individuals that did not appear to correlate directly with total or mean daily distances run. Figure 5 compares the histology of the quadriceps muscle from a control mouse (Fig. 5A), with exercised mouse 6 (Fig. 5B) and mouse 7 (Fig. 5C). While both exercised mice clearly show increased quadriceps necrosis compared with the sedentary control, it is notable that they ran significantly different total and mean daily distances (see Fig. 1A-B). The extent of variability in gastrocnemius muscle necrosis was also evident, with some mice (e.g. mouse 6) showing a similar level of necrosis to the control samples (compare Fig. 5D and E), and others (e.g. mouse 7) having extensive necrosis (compare Fig. 5D and F).

**Figure:**
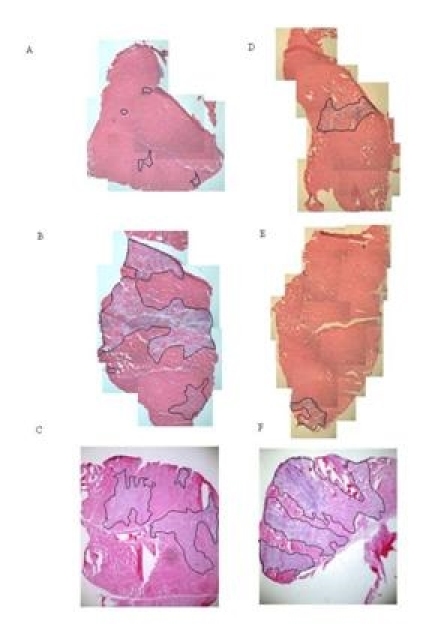
Figure 5: Images showing low-power, whole cross-sections through the quadriceps (A, B, C) and gastrocnemius (D, E, F) muscles from control mice (A, D), and exercised mice 6 (B, E), and 7 (C, F). The overlaid outlined areas indicate necrotic regions. The quadriceps muscles from exercised mice in general showed a higher percentage of necrotic tissue than in control samples (compare B and C with A). However, there was no obvious relationship between quadriceps pathology with the total distances run by mice, with mouse 6 (B) running >50 km, and mouse 7 (C) running <5km in total, yet showing similar extents of tissue necrosis. Gastrocnemius pathology was highly variable between exercised mice (compare E and F) and similarly did not obviously correlate with total distances run.  

As there was clearly high variability in quadriceps and gastrocnemius muscle histopathology and running behavior, we conducted a detailed analysis to determine if histopathology correlated with indices of running behaviour that were calculated across all nights of the study (Table 1). Three key running parameters showed a significant correlation with the percentage of tissue necrosis in the quadriceps muscle. The mean distance covered in individual running bouts showed a positive correlation, while the total number of running bouts, both throughout the entire study, and the mean number of bouts per night, correlated inversely, with tissue necrosis in the quadriceps muscle (Table 1). The mean nightly and total number of running bouts also showed a strong negative correlation with necrosis in the gastrocnemius muscle (Table 1). The mean running bout distance also correlated with the percentage of centrally nucleated myofibers (Table 1). Interestingly, parameters of rest time also correlated with necrosis in the gastrocnemius muscle, with total and mean rest times showing a positive correlation, and conversely, total run time having a negative relationship (Table 1). The only running parameter that was a significant positive indicator for myofiber CSA was the total cumulative rest time (Table 1). There were no significant correlations between any running parameters and either central nucleation or myofiber CSA in the gastrocnemius muscle (data not shown).   

 Table 1: Correlation coefficients between histopathological parameters (% central nucleation, % necrosis, and myofiber cross-sectional area (CSA)) in the quadriceps muscles from exercised mdx mice, and parameters of running performance. Correlations between % necrosis and running parameters are also shown for the gastrocnemius muscle. Significant relationships are emphasized in bold italics (p-values are indicated in parentheses (NS = not significant), n=13 in all cases). No significant correlations were observed for the gastrocnemius % central nucleation and CSA with any running parameters (data not shown).  

**Table:** 

	% necrosis		% central nucleation	myofiber CSA
	Quadriceps	Gastrocnemius	Quadriceps	Quadriceps
Cumulative total distance^a^	0.408 (NS)	-0.227 (NS)	0.521 (NS)	-0.335 (NS)
Mean daily distance	0.450 (NS)	-0.189 (NS)	0.551 (NS)	-0.338 (NS)
Total run time^a^	-0.037 (NS)	***-0.570*** (p=0.042)	0.217 (NS)	-0.288 (NS)
Mean run bout time^b^	0.399 (NS)	0.312 (NS)	0.480 (NS)	-0.351 (NS)
Mean run bout distance	***0.578 ***(p=0.039)	-0.082 (NS)	***0.623*** (p=0.023)	-0.242 (NS)
Total run bouts^a,c^	***-0.606 ***(p=0.028)	***-0.797*** (p=0.001)	-0.247 (NS)	-0.195 (NS)
Mean number of run bouts per night	***-0.625*** (p=0.022)	***-0.841*** (p=0.0003)	-0.340 (NS)	-0.275 (NS)
Mean run rate	0.336 (NS)	-0.297 (NS)	0.457 (NS)	-0.405 (NS)
Maximum run rate	-0.102 (NS)	-0.409 (NS)	0.292 (NS)	0.303 (NS)
Total rest time^a^	0.130 (NS)	***0.632*** (p=0.020)	0.052 (NS)	***0.571 ***(p=0.032)
Mean rest time	0.319 (NS)	***0.864*** (p=0.0001)	0.055 (NS)	0.521 (NS)

^a^Total values are cumulative values for all nights of the study. ^b^A run bout was deemed as any time during which mice generated wheel revolutions for at least two consecutive recording periods. ^c^The total and mean number of run bouts are also equivalent to the total and mean number of rest (non-wheel running) periods.   

Since the percentage of tissue necrosis specifically indicates recent acute muscle damage [20], we conducted a further correlation analysis on muscle necrosis and mouse running behaviour on each of the 3 days prior to tissue sampling (Table 2). The results of this analysis were highly consistent with those across the entire study (shown in Table 1) whereby there was a significant inverse relationship between the number of running bouts and tissue damage (Table 2). This was most striking (r_xy_ = -0.650; p=0.02) for the number of running bouts on the day before tissue sampling (Table 2). A corresponding analysis for the gastrocnemius muscles showed that the number of running bouts and the mean rest time similarly correlated strongly with the percentage of necrosis on each of the last three days prior to tissue sampling (Table 3). This was extremely strong for the mean rest time (Table 3).   

Table 2: Correlation coefficients between the percentage of necrosis (acute recent damage) in the quadriceps muscles from exercised mdx mice, and parameters of running performance on the last three days prior to tissue sampling. Significant relationships are emphasized in bold italics (p-values are indicated in parentheses (NS = not significant), n=13 in all cases). 

**Table:** 

	-1 day	-2 days	-3 days
Total distance^a^	0.030 (NS)	0.088 (NS)	0.365 (NS)
Total run time^a^	-0.411 (NS)	-0.361 (NS)	-0.111 (NS)
Mean run bout time	0.184 (NS)	0.256 (NS)	0.397 (NS)
Number of run bouts^b,c^	***-0.650*** (p=0.016)	***-0.618*** (p=0.024)	***-0.569*** (p=0.042)
Mean run bout distance	0.467 (NS)	0.442 (NS)	***0.630*** (p=0.021)
Mean run rate	0.256 (NS)	0.354 (NS)	0.450 (NS)
Mean rest time	***0.572*** (p=0.041)	0.185 (NS)	0.293 (NS)

^a^Total values are cumulative values for each night. ^b^A run bout was deemed as any time during which mice generated wheel revolutions for at least two consecutive recording periods. ^c^The number of run bouts are also equivalent to the total and mean number of rest (non-wheel running) periods.   

Table 3: Correlation coefficients between the percentage of necrosis (acute recent damage) in the gastrocnemius muscles from exercised mdx mice, and parameters of running performance on the last three days prior to tissue sampling. Significant relationships are emphasized in bold italics (p-values are indicated in parentheses (NS = not significant), n=13 in all cases).

**Table:** 

	-1 day	-2 days	-3 days
Total distance^a^	-0.227 (NS)	-0.190 (NS)	-0.345 (NS)
Total run time^a^	-0.528 (NS)	-0.510 (NS)	-0.360 (NS)
Mean run bout time	-0.185 (NS)	-0.265 (NS)	-0.361 (NS)
Number of run bouts^b,c^	***-0.641*** (p=0.018)	***-0.675*** (p=0.011)	***-0.793*** (p=0.001)
Mean run bout distance	0.040 (NS)	-0.050 (NS)	-0.092 (NS)
Mean run rate	-0.230 (NS)	-0.186 (NS)	-0.196 (NS)
Mean rest time	***0.867*** (p=0.0001)	***0.836*** (p=0.0004)	***0.883*** (p=0.00006)

^a^Total values are cumulative values for each night. ^b^A run bout was deemed as any time during which mice generated wheel revolutions for at least two consecutive recording periods. ^c^The number of run bouts are also equivalent to the total and mean number of rest (non-wheel running) periods.  

## Discussion

The results of this study clearly demonstrate extensive individual variation in voluntary wheel running in adult mdx mice. We also observed considerable variability in histopathological parameters, as indicated by high standard deviations on mean values. Therefore, we conducted a detailed analysis of running parameters and their relationship to muscle pathology. In the quadriceps muscle, the strongest determinants of disease phenotype exacerbation were the number and distance of running events, whereby fewer running bouts, and higher bout distance, promoted muscle damage.  A similar relationship existed for necrosis in the gastrocnemius. As the number of running bouts also equates with the number of rest breaks (non-running periods), it can also be extrapolated that a low number of rest breaks correlates with increased muscle damage. This is further supported by the observation that the total cumulative rest time correlated with myofiber cross-sectional area, which suggests a greater rest time leads to sufficient tissue recovery and the regeneration/growth of newly formed myotubes. In the gastrocnemius muscle, there was also a very strong positive correlation between necrosis and the mean rest times. While it is seemingly paradoxical that long periods of rest would promote muscle damage, one possibility is that long periods of inactivity have a "cool down" effect on the muscle, making it more susecptible to acute injury at the onset of an exercise bout. While there is evidence that longer periods of rest (i.e. >1 week) lead to re-injury of muscle tissue once wheel running resumes [21], there are no studies that we are aware of that examine the impact of more regular, or longer, rest breaks between running bouts. Taken together, these data strongly indicate that a detailed analysis of individual mouse running behavior, with a particular focus on the number of run/rest cycles, and the distance covered in individual running bouts, is essential when utilizing voluntary wheel running to exacerbate the dystrophic phenotype in the mdx mouse. It also demonstrates some variability in the running parameters that impact on the disease phenotype in different muscle groups. Interestingly, many studies to date utilizing exercise wheels to examine either disease pathogenesis or the efficacy of therapeutic regimes have relied on examining mean daily or cumulative distances run [15] [22]. Our data show that while these parameters do correlate with histopathology to a certain extent, they were not the strongest indicators.   

Our data are in strong accordance with previous studies that reported extensive individual variation in voluntary wheel running in mdx mice [9] [17]. Interestingly, we observed a striking increase in individual variability, rather than a convergence or equalizing of performance over time, in the range of daily distances run by individual animals increasing progressively throughout the trial. Such individual variation may also explain the controversy in the literature, with some studies reporting detrimental effects of this form of exercise on mdx dystropathology [11] [16], while others have shown an improvement in various aspects of the dystrophic phenotype [12] [14] [15]. Exercise regimes can similarly be deleterious or detrimental to muscle structure and function in humans with DMD, depending on the design of the exercise program [23] [24]. Our data suggest that in mdx mice exercise regimes that allow for a low number of low intensity exercise bouts are most likely to minimize muscle damage. As murine data cannot directly be extrapolated to the human disease, further human-based studies are required to determine if similar aspects of exercise behaviour impact on muscle damage.   

In the present study, three key aspects of muscle histopathology were examined. The percentage of tissue necrosis, indicating recent muscle fiber damage followed by degeneration and inflammation, increased approximately 2-fold in the quadriceps muscles from exercised mdx mice, which is similar to that previously reported for mdx mice with voluntary wheel access for only 48 hours [9] [17]. This raises the issue of how long mice require wheel access for before an impact will be observed, and indeed our correlation studies on running behaviour in the three days prior to tissue sampling indicate that up to 72 hours of wheel exposure causes significant acute muscle damage. This was the case for both the quadriceps and gastrocnemius muscles. Longer term studies are required to determine if prolonged wheel running has a similar effect in mdx mice as that observed in some wild type mouse strains, whereby initial damage (within the first two weeks) is sustained, but long-term use has little or no detrimental effect [25]. Initial wheel use-induced damage similarly occurs in periodic wheel running interspersed with rest periods of 3 weeks [21].  Thus, where acute tissue damage is utilized as a histopathological indicator in the mdx mouse model only brief exposure to running wheels is required for an impact to be observed. It is as yet unclear if longer term wheel and periodic wheel running has a similar impact in mdx mice as in certain wild-type strains, and further research on a range of running patterns and protocols is required to clarify these issues.   

Myofiber cross-sectional area was, as expected, highly variable between both muscle and treatment groups, which is likely to result from cyclic degeneration and subsequent repair in dystrophic muscle. Exercise did not have a significant effect on mean myofiber size in either the quadriceps or gastrocnemius muscles, although for both muscle groups exercise did cause a slight shift from small (<500µm^2^) to larger (>1000µm^2^) myofibers. This suggests that voluntary wheel running promotes the growth of very small, newly formed myofibers in both the quadriceps and gastrocnemius muscles. This might be particularly the case for the gastrocnemius muscles in which exercise also caused a significant improvement in tissue necrosis as discussed above. The gastrocnemius muscles from both control and exercised mdx mice showed a 2-fold increase in the presence of both very small and very large myofibers compared with the quadriceps muscles, and this is consistent with previous studies that reported both a shift towards larger myofibers [26] and the preservation of very small myofibers [27] in this muscle group in mdx mice between the ages of 2 and 6 months (which encompasses the mice used in the present study). It is noted that in the present study, exercised mice had wheel access for an acclimatization and calibration period prior to the commencement of the data collection period. It is possible that some individuals utilized the wheels more than others during this time, and that this may have impacted on the myofiber cross-sectional area data, and further studies that incorporate larger sample sizes and more controlled wheel access are required to more clearly resolve the impact of exercise on fiber size. Given that small areas of acute tissue damage is typically resolved within 5-7 days, it is not expected that the acclimatization period would have impacted on the tissue necrosis data in the present study.   

In summary, we have utilized a voluntary exercise wheel design that collects wheel use data (in revolutions) at regular intervals, to demonstrate the complexity of the impact of intermittent and self-moderated exercise on muscle pathology in mdx mice. Of key importance is the need to analyze the number and distance covered in exercise bouts, and to select the most appropriate muscle group/s for analysis. 

## Acknowledgements

 The authors would like to thank Mr Robert White for wheel and software design and development and extensive technical assistance.    

## Funding information

This research was funded by a research grant from the Association Francaise contre les Myopathies (GMS, project ID 13211), and from the Muscular Dystrophy Association (Australia) to JDW.

## Competing Interests

The authors have declared that no competing interests exist.
